# Plant defence priming and memory mechanisms under climate change

**DOI:** 10.1042/EBC20250050

**Published:** 2026-07-30

**Authors:** Rosa Sanchez-Lucas

**Affiliations:** 1 School of Biosciences, University of Birmingham, Birmingham B15 2TT, U.K.; 2 Birmingham Institute of Forest Research, University of Birmingham, Birmingham B15 2TT, U.K.

**Keywords:** climate change, plant memory, plant stresses, priming of defence

## Abstract

Climate change exposes plants to increasingly variable and overlapping abiotic and biotic stresses, challenging ecosystem stability as well as the survival and productivity of crops beyond the limits of classical defence strategies. Defence priming, defined as the capacity of plants to respond faster, stronger, or more effectively following prior exposure, has emerged as a key adaptive mechanism and a promising strategy to enhance plant protection and ecosystem resilience under recurrent or combined stress conditions. Here, current understanding of priming and defence memory in the context of climate change is synthesised, focusing on four interrelated dimensions: (1) the signalling networks that encode and transmit priming, (2) epigenetic and transcriptional mechanisms underpinning somatic and transgenerational memory, (3) physiological and fitness trade-offs associated with primed states under altered temperature, drought, and elevated CO_2_, and (4) experimental evidence demonstrating how climate-related abiotic factors modulate priming outcomes and plant-pathogen interactions across spatial and temporal scales. Recent studies reveal that climate drivers can both enhance and constrain priming, depending on stress combinations, timing, and intensity. Understanding the mechanisms that determine these context-dependent outcomes will be critical for predicting when priming can be effectively deployed to enhance crop resilience, sustain productivity, and reduce reliance on conventional pesticides under ongoing climate change.

## Introduction

**Figure 1:**
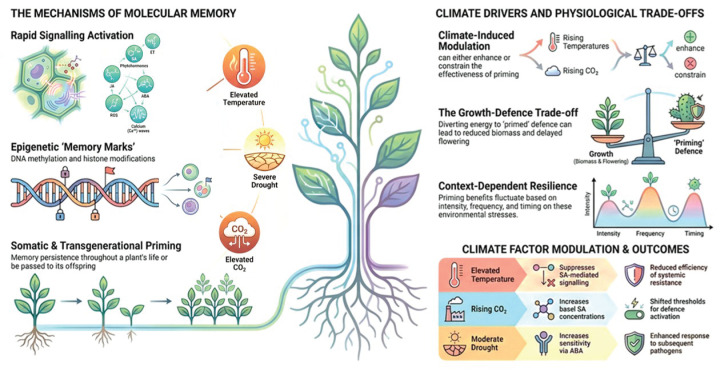
Climate-shaped defence priming drives plant resilience under combined stress Environmental stress history, microbial partners, and epigenetic memory interact to determine whether priming enhances or constrains plant defence under climate change because of imbalance in biochemical processes. Image drawn with NotebookLM and modified using Inkscape software v 1.4.2.

Climate change is reshaping the environmental landscape in which plants grow, increasing the frequency and variability of abiotic stresses while altering/intensifying interactions with pathogens and herbivores, producing complex and overlapping stress scenarios rather than isolated pressures. Increasingly, experimental and field studies show that plant responses to biotic stress are strongly influenced by prior or concurrent exposure to abiotic factors, often in non-additive and context-dependent ways [1–5]. These interactions challenge traditional models of constitutive resistance or single-stress optimization. Within this context, the escalating impact of climate change on plant health and ecosystem stability underscores the urgent need for effective mitigation and adaptation strategies. Advances in plant biological and molecular knowledge, together with their biotechnological translation, are expected to play a pivotal role in strengthening plant resilience [6] (Figure 1).

**Figure 2:**
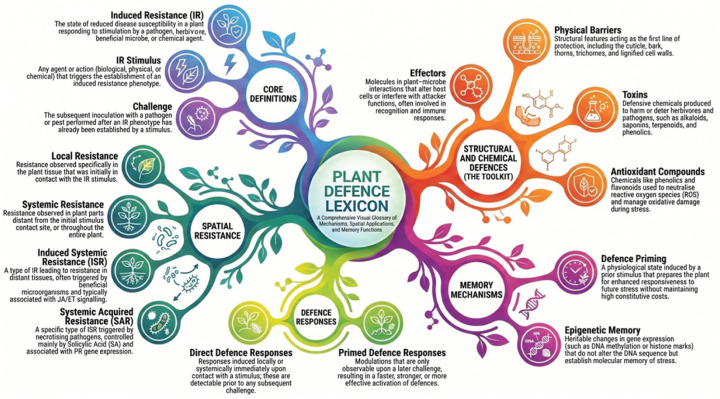
Lexicon of defence priming and memory in plants Schematic infographic providing a glossary of key terms used throughout the present review. The lexicon summarises core concepts in plant defence priming, signalling networks, epigenetic regulation, and stress memory, including their roles in mediating responses to combined abiotic and biotic stress under climate change. The glossary differentiates between direct defence responses, where elicitors trigger immediate activation of defence pathways (e.g., defence gene expression, PR protein accumulation, ROS production, or localised cell death), and priming, which establishes a sensitised physiological state without necessarily inducing substantial defence deployment until a subsequent challenge occurs. Definitions are aligned with current usage in plant stress biology and are informed by established frameworks of induced resistance and priming terminology [11]. Image drawn with NotebookLM and modified using Inkscape software v 1.4.2.

Although constitutive defence activation can provide immediate protection, it often incurs substantial fitness costs under resource-limiting or fluctuating environments [7,8]. To balance protection with growth and reproduction, plants rely heavily on inducible strategies, among which defence priming has emerged as a central mechanism [9]. Priming, originally coined in the context of plant immunity and later extended to abiotic stresses, can be defined as a plant physiological state induced by a prior physical, chemical, or biological stimulus that triggers plants for a faster or stronger response upon subsequent challenge [10]. Thus, rather than maintaining defence mechanisms constitutively, primed plants remain in a heightened state of alert, enabling faster activation of stress-responsive genes when needed. This strategy is particularly advantageous under fluctuating and recurrent stress scenarios, as it minimises fitness costs while maximizing adaptive capacity. To improve accessibility for readers new to the field, Figure 2 includes a brief lexicon summarising key defence priming and induced-resistance terminology, adapted from De Kesel et al. [11].

A wide range of stimuli can trigger priming in plants, including biotic cues such as pests and pathogens, as well as beneficial microorganisms like rhizobacteria and mycorrhizal fungi [12–15]. Various chemical compounds can also act as priming agents, including phytohormones (e.g. salicylic acid (SA), jasmonic acid (JA), and ethylene (ET)) [16], synthetic molecules such a beta-aminobutyric (BABA) [17], elicitor extracts [18], and plant emitted volatiles that induce priming in neighbouring undamaged plants [19–21].

Importantly, primed states can persist beyond the initial stimulus, in some cases throughout plant development or even across generations, highlighting their central role in stress memory and adaptive plasticity [22,23]. In the context of climate change, this memory is particularly significant, as plants are increasingly exposed to overlapping, recurrent, and unpredictable stress combinations. Growing evidence indicates that persistence of primed states is closely associated with epigenetic mechanisms (DNA methylation, histone modifications, and small RNA pathways) that establish molecular memory marks that modulate gene expression [24]. Understanding how epigenetic processes shape the establishment, maintenance, and resetting of primed states is therefore essential for elucidating plant resilience under climate change scenarios. Yet climate change does more than increase the importance of priming; it reshapes the context in which priming is established, maintained, and expressed. Heat, drought, and elevated CO_2_ can alter signalling pathways, hormonal cross-talk, and resource allocation, thereby modifying the effectiveness, costs, and persistence of primed states [25–29] (Figure 1).

In the present review, we synthesise current knowledge on defence priming as a mechanism of plant defence (Figure 2) and adaptation under changing environments. We focus on signalling and metabolic networks, epigenetic memory mechanisms, physiological trade-offs, and experimental evidence of climate modulation. By integrating mechanistic insights with climate-relevant stress scenarios, we aim to clarify when, how, and under what conditions priming contributes to plant resilience in our rapidly changing world.

## Priming signalling and its modulation by climate drivers

Defence priming relies on a complex network of signalling pathways that integrates local stress perception with systemic communication and transcriptional and metabolic adjustments, preparing plants for enhanced responsiveness. Central to this network are phytohormones, including SA, JA, ET, and abscisic acid (ABA), as well as reactive oxygen species (ROS), calcium (Ca^2+^) waves, and mobile molecular signals that coordinate responses across tissues deeply changing the secondary metabolism [30–32]. These elements interact dynamically, providing both stimulus specificity and flexibility to respond to recurring or combined stress events [1,33].

SA is pivotal in priming against biotrophic pathogens. In systemic acquired resistance (SAR), pathogen detection locally triggers SA accumulation, amplifying defence gene expression in distal tissues and establishing a primed state [34,35]. JA and ET mediate defences against necrotrophs and herbivores, often interacting antagonistically or synergistically with SA [1,19,20,36]. ABA serves as a bridge between abiotic and biotic stress signalling, modulating the amplitude and duration of primed responses to drought or osmotic stress [9,37]. Early events in priming are mediated by ROS and Ca^2+^ waves, which rapidly propagate through plant tissues to activate transcriptional regulators and potentiate systemic defence readiness [30,32]. Collectively, these networks maintain low basal defence activity under non-stress conditions while allowing rapid, robust responses when needed.

Priming can be induced by diverse stimuli, including pathogen attack, herbivory, beneficial microbes, chemical elicitors, and abiotic stresses. Locally, prior exposure sensitises signalling components, resulting in faster activation of defence genes upon subsequent challenge. Systemically, mobile signals (including peptides, lipid-derived molecules, and small RNAs) transmit information to distant tissues, establishing a whole-plant primed state [10,12,38]. For example, chromatin modifications, such as H3K4me3 deposition at defence loci, regulate genes for rapid induction without constitutive activation, providing a mechanistic basis for the persistence of priming [39,40].

Climate-related abiotic factors strongly influence these signalling networks. Temperature is a particularly important determinant of SA-mediated priming. Elevated temperatures can suppress SA biosynthesis and/or its downstream signalling, reducing the efficiency of SAR [41,42], while mild heat or chilling can sometimes enhance SA-dependent priming [25,42], highlighting the context dependence of temperature effects and threshold temperatures in physiological and phenological processes. JA- and ET-mediated pathways are similarly sensitive to temperature fluctuations, with high heat occasionally impairing JA accumulation and defence gene expression, especially under combined abiotic and biotic stress [43,44]

Regarding rising CO_2_ atmospheric concentration, elevated CO_2_ can increase basal SA concentrations in Arabidopsis and tobacco, affecting the thresholds for defence gene activation and the kinetics of primed responses [26]. This shift can interact with hormonal cross-talk, for example by altering JA-SA antagonism, thereby modifying priming outcomes under combined stresses [45]. Elevated CO_2_ can also influence stomatal conductance and ROS signalling, indirectly affecting both local and systemic priming. With other priming stimuli such as BABA, elevated CO_2_ caused a reduced protection in oak-powdery mildew interaction under controlled growth cabinet conditions [27].

Drought and osmotic stress, primarily mediated by ABA, modulate priming as well. Moderate drought can enhance primed responses, increasing sensitivity to subsequent pathogen attack, whereas severe water limitation can suppress defence gene induction, likely reflecting resource prioritisation under extreme stress [46–49]. Importantly, the sequence and combination of stresses determine net outcomes: pre-exposure to moderate heat or drought can either synergise with pathogen-induced priming or interfere with it, depending on timing and compatibility [43,47,50,51]. Additionally, exposure to heat followed by drought may activate different signalling and metabolic trajectories than the reverse sequence [52]. All these interactions illustrate that priming under climate change is highly context-dependent, shaped by temperature, water availability, and CO_2_ levels, where the sequence-dependent effects remain essential for predicting plant resilience under future climate scenarios.

ROS and Ca^2+^ signalling, key early mediators of priming, are themselves sensitive to environmental fluctuations. Heat and drought can accelerate ROS production, which may enhance or disrupt systemic signalling depending on antioxidant capacity [32,53,54]. Ca^2+^ waves, propagating stress information to distal tissues, are similarly modulated by temperature and water status, affecting the speed and intensity of primed responses [30,55,56]. Collectively, these findings underscore that abiotic conditions can directly influence the signalling circuits underlying priming, shaping both local and systemic defence readiness.

Beyond direct plant–pathogen interactions, volatile organic compounds mediate indirect defence responses by recruiting natural enemies of herbivores and pathogens, forming part of complex multitrophic signalling networks. These interactions are increasingly recognised as sensitive to environmental conditions and may be disrupted or reshaped under climate change, potentially altering the effectiveness of indirect defence priming [51].

Overall, priming emerges as a dynamic, environment-sensitive state rather than a fixed trait. Temperature, drought, and CO_2_ fluctuations alter basal hormone levels, signalling sensitivity, and systemic communication, ultimately determining the magnitude and persistence of primed responses. Deciphering these interactions is essential to predict defence outcomes under climate change and to strategically exploit priming for crop resilience (Figure 1). Especially considering how this also feed into epigenetic modifications that encode memory, ensuring that plants retain readiness across developmental stages and, in some cases, generations and how the predicted climate factors will change several interactions and distribution of microorganism a present on the soil that act as induced resistance stimuli [57].

## Epigenetics mechanisms of defence memory and its modulation by climate change

Epigenetic mechanisms, defined as heritable, reversible, and plastic changes in gene expression without altering the DNA sequence, are central to how plants retain and recall information from previous stress encounters. DNA methylation, histone modifications, and chromatin remodelling contribute to both somatic stress memory and, in some cases, transgenerational priming [22,24,58,59]. Unlike transient transcriptional activation, epigenetic modifications can persist through cell divisions, providing a biochemical basis for memory that shapes future responses. The role of these modifications, particularly cytosine methylations, has been associated with silencing and/or activation of genes and transposable elements (TEs), where CHH methylation is closely linked to small RNA-directed DNA methylation pathways. This is particularly relevant because TE activation can contribute to stress responses and adaptive variation under changing environmental conditions [59]. Furthermore, a substantial proportion of stress-induced methylation changes occur within TE-rich regions of plant genomes [60], suggesting that climate-associated alterations in epigenetic regulation may influence both defence memory and genome plasticity. Repeated exposure to abiotic stresses such as drought or temperature extremes can induce dynamic DNA methylation changes that correlate not only with TE’s but also with altered stress-responsive gene expression. In *Medicago ruthenica*, drought triggers genome-wide hypomethylation, enhancing expression of ABA and proline biosynthesis genes, thereby amplifying subsequent drought responses [59]. Similar patterns are observed in rice and other crops, where drought induces methylation or demethylation at specific loci, with some marks persisting after stress to create memory-like transcriptional states [3,60,61]. These modifications modulate transcriptional responsiveness, allowing plants to prioritise stress responses when future challenges arise. In addition, high temperatures can induce both hyper- and hypomethylation at stress-related loci in crops such as *Brassica napus* and *Setaria italica*, often in a genotype-specific manner [62,63]. In *Arabidopsis thaliana*, variation in -CHG and CHH methylations respond to environmental temperature gradients, suggesting that temperature modulates RdDM and chromomethylase activity [64], which in turn influences the stability of priming-associated epigenetic marks.

Epigenetic regulation interacts with hormonal signalling to link climate stress with defence memory [40,65]. During drought and heat stress, ABA-dependent signalling can reshape DNA methylation patterns at stress-responsive loci, facilitating faster transcriptional activation upon subsequent challenge. These modifications, occurring in CG, CHG, or CHH sequence contexts, may persist after stress recovery and establish a primed transcriptional state. Moreover, epigenetic effects can also extend to transgenerational priming. Pathogen-induced defence states can be transmitted to offspring, with primed progeny showing elevated defence gene induction [66–68]. In *Arabidopsis*, mutations in RdDM components disrupt the transmission of SA-dependent primed responses, implicating DNA methylation as a carrier of memory across generations [66]. Climate-associated stresses experienced by parents may therefore reshape progeny epigenomes, influencing adaptive outcomes. However, the stability and ecological relevance of these effects remain context-dependent, as some methylation marks are reset during gametogenesis or early development [24,58,69].

Epigenetic mechanisms provide a biochemical substrate for somatic and, in some cases, transgenerational priming. Climate factors such as recurrent drought and temperature extremes can reshape these methylation landscapes, modulate stress-responsive networks, and influence the persistence and effectiveness of primed states, with important implications for resilience in natural and agricultural systems.

## Trade-offs defence mechanisms and epigenetic memory under climate change

While defence priming and epigenetic memory confer adaptive advantages, they are not without cost. Maintaining a primed state requires allocation of energy and metabolites, which may reduce resources available for growth, reproduction, or other physiological processes [46,70–72]. These phenotypic trade-offs are particularly pronounced under the variable and extreme conditions imposed by climate change, where the fitness benefits of priming may fluctuate depending on stress type, intensity, frequency, and resource availability. To complement this, evolutionary trade-offs may arise when genetic variation underlying defence inducibility is negatively associated with growth or constitutive resistance strategies, although the extent to which such relationships influence priming remains poorly understood [73].

Studies demonstrate that maintaining a primed state can limit growth under non-stress conditions. Arabidopsis plants primed for SA-dependent defence show reduced biomass and delayed flowering, reflecting a shift in carbon and nitrogen allocation from growth to defence [9,10]. Similarly, JA-mediated priming against herbivory can reduce leaf area and reproductive output [72]. These examples highlight the context-dependence of priming: benefits must be weighed against potential growth penalties, especially when stresses are intermittent or unpredictable.

Epigenetic mechanisms can amplify these trade-offs. Persistent methylation at stress-responsive loci may prime gene expression at the cost of metabolic flexibility, particularly under fluctuating environments [22,24,60,63]. Drought-induced methylation in rice and Arabidopsis enhances subsequent stress tolerance but can reduce growth under favourable conditions [3,60,61]. Transgenerational priming can protect progeny against pathogens or abiotic stress but may also reduce seed size, germination, or early growth if the primed state is mismatched to environmental conditions [58,66,67,69].

Climate-change factors further influence the cost-benefit balance. Heat stress can exacerbate growth penalties in SA-primed plants [14], while drought-primed plants may exhibit enhanced ABA responses but reduced carbon assimilation under high evaporative demand [1,2,37]. Elevated CO_2_ can offset some growth costs by increasing carbon availability, though benefits depend on stress congruence [26,27,45]. Stress predictability and frequency also shape trade-offs. Frequent and predictable stress favours priming and epigenetic memory, enabling faster, stronger responses without continuous activation costs [8,70,71]. Conversely, in highly variable or novel climates, priming may divert resources from growth when stress does not occur. These dynamics underscore the adaptive significance of plasticity, with plants capable of modulating priming magnitude and duration likely outperforming rigid strategies under climate change.

From an applied perspective, understanding these trade-offs is essential. Transient or stimulus-specific priming, genotype selection, and optimized timing can mitigate costs while maintaining defence benefits. Integrating climate-responsive priming traits into breeding programs could enable crops to anticipate recurrent stresses without compromising yield under favourable conditions. This takes critical relevance under the increasingly unpredictable stress regimes imposed by climate change, where both phenotypic and evolutionary trade-offs may determine whether priming can incur fitness costs that outweigh its defensive benefits.

## Future perspectives

Harnessing priming and epigenetic memory offers a path towards crops that can anticipate recurring stresses, maintain yield under extreme conditions, and adapt to the unpredictable realities of a changing climate.

Despite the considerable advances in understanding plant priming, epigenetic memory, and the associated growth-defence trade-offs, several critical gaps remain that constrain our ability to translate these insights into crop improvement under climate change (Figure 1). One major limitation is that most mechanistic studies have examined stresses in isolation. Although extensive mechanistic insights from single-stress studies exist, our ability to predict priming outcomes under the dynamic, multi-stress environments imposed by climate change remains limited, a critical barrier to translating knowledge into resilient crops. In natural and agricultural environments, however, plants rarely encounter a single stress; they are more often subjected to combinations of abiotic and biotic challenges, such as heat waves coinciding with drought or pathogen outbreaks. Integrative studies that capture the complexity of fluctuating, multi-stress environments are still scarce, leaving a gap in our understanding of how priming and memory operate under realistic conditions [1,47,48,50,74].

Another gap concerns the causal links and stability of epigenetic modifications. While DNA methylation and histone marks are frequently associated with priming and stress memory (Figure 2), direct evidence connecting specific modifications to phenotypic stress tolerance is still limited, particularly when considering transgenerational inheritance. The field requires more studies that move beyond correlations to mechanistically establish which epigenetic marks are both necessary and sufficient for enhanced tolerance [58]. Closely related to this is the temporal dynamics and persistence of primed states. Somatic memory, transgenerational effects, and the reversibility of epigenetic modifications remain incompletely characterized, especially under variable environmental conditions. Without this knowledge, it is difficult to predict how long a primed state will persist, how it might interact with subsequent stresses, or whether it will be beneficial or costly under changing conditions.

An important challenge is the quantification of trade-offs under realistic field conditions. Growth-defence trade-offs, a key constraint on the utility of priming, have mostly been documented in controlled laboratory or greenhouse environments [25,30,44,70]. The complexity and variability of field conditions (fluctuating temperature, water availability, and pathogen pressure) can profoundly alter the costs and benefits of priming, yet these effects remain largely unquantified [46,71,72].

Another underexplored dimension is the influence of ontogeny on defence priming and memory. Priming capacity, inducibility, and associated fitness costs can vary across developmental stages, reflecting shifts in resource allocation and defence priorities throughout the plant life cycle. Evidence from wild radish demonstrates that inducible defences, transgenerational induction, and transgenerational priming can differ markedly among life stages, suggesting that plant age may be an important determinant of priming effectiveness under changing environments [75].

Finally, our understanding of genotype and environment interactions in priming is still limited. Most research has focused on model species such as *Arabidopsis thaliana* or a small number of crop species, leaving open questions about how natural genetic variation shapes the magnitude, duration, and plasticity of priming responses across diverse climates [9,25,68,69,76]. Interestingly, a previous study associated natural varietal resistance with the ability to mount inducible defences, with susceptible individuals exhibiting a stronger response to priming stimuli [77].

Addressing these gaps will require integrative experimental designs that combine high-resolution omics approaches, detailed epigenetic mapping, physiological phenotyping, and environmental monitoring across both controlled and field conditions. Looking forward, future research should prioritise several complementary directions. Multi-stress studies that simultaneously evaluate abiotic and biotic challenges will provide a more realistic understanding of priming under climate-change scenarios. In addition, the application of high-resolution epigenomics, such as bisulphite sequencing, long-read sequencing platforms (e.g. Oxford Nanopore and PacBio), chromatin immunoprecipitation, and single-cell approaches, will be essential to map specific methylation and histone marks that drive stress memory [22,24,78]. Moreover, investigations of transgenerational plasticity are needed to determine the heritability and reversibility of epigenetic priming, including its fitness consequences for progeny exposed to variable environments [67].

To translate mechanistic insights into practical outcomes, model-to-field approaches are critical. Crop trials in realistic environmental conditions can quantify trade-offs, yield stability, and resilience under combinations of heat, drought, and elevated CO_2_ [17,25]. Simultaneously, breeding and biotechnological strategies that incorporate knowledge of epigenetic priming, such as selecting genotypes with flexible, context-sensitive memory mechanisms, could enhance climate-resilient crops. Finally, integrative modelling frameworks that link molecular, physiological, and climate data can predict the cost-benefit balance of priming and memory under future scenarios, guiding both research and applied management decisions [79]. By integrating mechanistic, ecological, and agronomic data, we can move from descriptive understanding towards predictive models of plant resilience.

## Conclusions

Defence priming and epigenetic memory represent powerful strategies that enable plants to anticipate, respond to, and recover from recurrent and variable stresses. By accelerating and amplifying defence responses, these mechanisms can enhance plant fitness under the frequent and extreme environmental challenges associated with climate change. However, their benefits are highly context-dependent, constrained by growth–defence trade-offs, environmental variability, and resource availability.

Climate drivers, including elevated temperatures, altered precipitation, and rising CO_2_, can modulate signalling, priming efficiency, and epigenetic stability, reshaping the cost-benefit balance of defence memory. Importantly, these factors influence both the magnitude and persistence of primed states but also the fitness outcomes, highlighting the need for integrative molecular, physiological, and environmental approaches.

Future research must bridge mechanistic insights with field-scale realities, combining high-resolution omics, epigenetics, phenotyping, and predictive modelling. Translating this knowledge into practical strategies (chemical, biological, and epigenetic priming, as well as breeding for flexible stress memory) offers the potential to design crops that anticipate recurrent stresses, maintain productivity under extreme conditions, and adapt to the unpredictable realities of a changing climate. Harnessing these mechanisms could be key for sustaining global agricultural and forestry resilience in a rapidly changing world.

## Summary

- Climate change exposes plants to increasingly variable and overlapping abiotic and biotic stresses, which can modify plant defence responses and plant–pathogen interactions.
- Defence priming allows plants to respond faster and more effectively after prior stress exposure, representing a potential mechanism to enhance resilience in both natural and agricultural systems.
- Priming responses are mediated by interconnected signalling networks and epigenetic mechanisms that encode stress memory at somatic and, in some cases, transgenerational levels.
- Understanding how climate drivers influence priming will help predict plant defence outcomes and guide strategies to improve crop resilience while reducing reliance on chemical pesticides.

## Open Access

Open access for this article was enabled by the participation of University of Birmingham in an all-inclusive Read & Publish agreement with Portland Press and the Biochemical Society under an agreement with JISC.

## Abbreviations

ABA: abscisic acid
BABA: beta-aminobutyric
Ca^2+^: calcium
ET: ethylene
H3K4me3: Histone H3 lysine 4 trimethylation
JA: jasmonic acid
ROS: reactive oxygen species
RdDM: RNA-directed DNA methylation
SA: salicylic acid
SAR: systemic acquired resistance
siRNAs: small interfering RNAs
TEs: transposable elements
